# Small bowel adenocarcinoma: natural history of recurrence after surgical resection

**DOI:** 10.1093/jscr/rjac451

**Published:** 2022-10-27

**Authors:** Massimo Farina, Francesco Falbo, Andrea Biancucci, Giorgio Lucandri, Vito Pende, Paolo Mazzocchi, Francescopaolo Cascini, Alessandro Lembo, Emanuele Santoro

**Affiliations:** UOC Chirurgia Generale ad Indirizzo Oncologico, Direttore Prof. Emanuele Santoro, Azienda Ospedaliera San Giovanni Addolorata, Roma, Italy; UOC Chirurgia Generale ad Indirizzo Oncologico, Direttore Prof. Emanuele Santoro, Azienda Ospedaliera San Giovanni Addolorata, Roma, Italy; UOC Chirurgia Generale ad Indirizzo Oncologico, Direttore Prof. Emanuele Santoro, Azienda Ospedaliera San Giovanni Addolorata, Roma, Italy; UOC Chirurgia Generale ad Indirizzo Oncologico, Direttore Prof. Emanuele Santoro, Azienda Ospedaliera San Giovanni Addolorata, Roma, Italy; UOC Chirurgia Generale ad Indirizzo Oncologico, Direttore Prof. Emanuele Santoro, Azienda Ospedaliera San Giovanni Addolorata, Roma, Italy; UOC Chirurgia Generale ad Indirizzo Oncologico, Direttore Prof. Emanuele Santoro, Azienda Ospedaliera San Giovanni Addolorata, Roma, Italy; UOC Chirurgia Generale ad Indirizzo Oncologico, Direttore Prof. Emanuele Santoro, Azienda Ospedaliera San Giovanni Addolorata, Roma, Italy; Direttore UO Oncologia Medica, Casa di Cura Marco Polo, Rome, Italy; UOC Chirurgia Generale ad Indirizzo Oncologico, Direttore Prof. Emanuele Santoro, Azienda Ospedaliera San Giovanni Addolorata, Roma, Italy

**Keywords:** small bowel, surgical oncology, recurrence, adenocarcinoma

## Abstract

Small bowel adenocarcinomas (SBA) are a rare entity associated with a poor prognosis and an advanced stage of disease at diagnosis. Surgical resection is considered the gold standard of treatment for stage I-III, while stage IV disease approach is still debated. We present a case of a young woman affected by a duodenojejunal junction SBA treated with surgical resection and FOLFOX adjuvant chemotherapy. The patient later underwent a palliative duodenojejunal bypass for peritoneal carcinomatosis.

## INTRODUCTION

On August 2020, National Comprehensive Cancer Network released the first edition of small bowel adenocarcinoma (SBA) treatment guidelines. These recommendations addressed and reinforced the differences in treatment and behavior between this rare entity and colorectal cancer, which were historically managed the same way.

Small bowel cancers are a relatively rare entity, accounting for only 3–5% of all gastrointestinal tumors, and 30–40% are SBA and over a half of these tumors arise in the duodenum [2]. Compared with other GI cancer, patients affected by SBA are usually younger and seek medical attention with an advanced stage of disease, due to the natural history of the tumor.

In this paper, we report a case of an SBA arising from the jejunum and its rapid progression after the first successful operation and adjuvant chemotherapy.

## CASE REPORT

**Figure 1:**
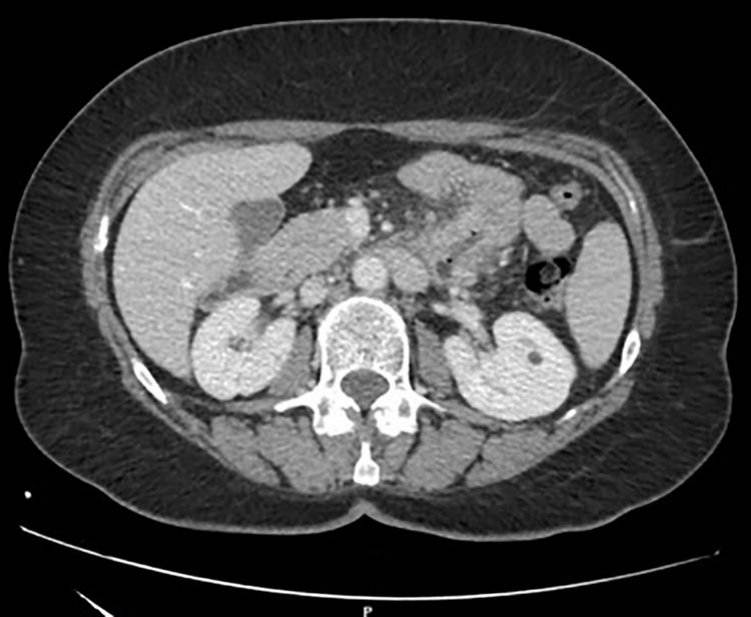
Contrast-enhanced CT scan.

**Figure 2:**
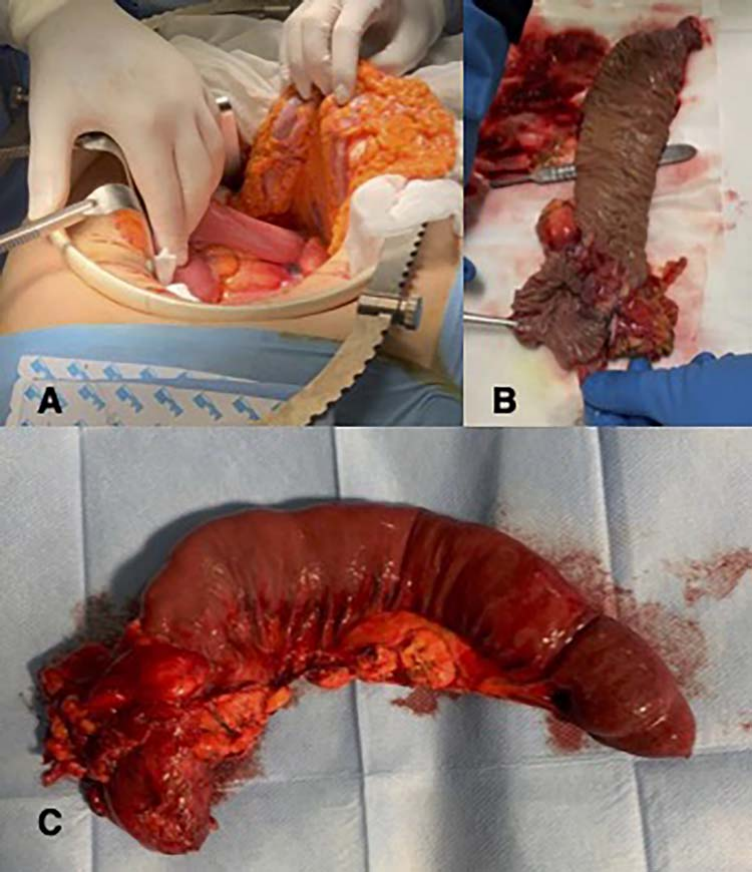
(**A**) Intra-operative image of duodenojejunal junction neoplasm; (**B** and **C**) histopatological examination.

A 57-year-old woman was admitted in our General Surgery department lamenting fatigue, diffuse abdominal pain and dyspepsia. The patient had no previous history of abdominal surgery or pre-existing conditions. On physical examination, there were no signs of abdominal masses or tenderness. Contrast-enhanced computed tomography showed a soft tissue mass of the duodenojejunal junction with initial signs of narrowing of the lumen (Fig. 1). We decided to perform a duodenojejunal resectionwith open approach. The tumor showed signs of extra-serosal extension and infiltration of the transverse mesocolon, the mesentery and the inferior mesenteric vessels. A complete abdominal exploration ruled out the localization of peritoneal carcinosis. Kocher maneuver was performed, and the tumor was resected en bloc with a portion of transverse mesocolon and mesenteric peritoneum. The intestinal continuity was restored with a linear stapled duodenojejunal side-to-side anastomosis (Fig. 2). The post-operative course was uneventful, and the patient was discharged on IX POD. Histological examination showed a poorly differentiated signet-ring cell adenocarcinoma of the small bowel, pT4 pN0 (0/19) pM1 (peritoneum) R0, L1, V1, Pn1, infiltrative tumor margin, tumor budding was present. During oncological follow-up, the patient was administered with 11 cycles of FOLFOX. Thirteen months later, the patient presented to the emergency department with recurrent episodes of vomiting and upper abdominal pain. A contrast-enhanced CT scan showed a relapse with intestinal obstruction. A re-laparotomy was performed; a fibrous and firm hernia sac was resected and sent to extemporaneous histopathological examination, showing localization of carcinomatosis. Diffuse miliary peritoneal carcinomatosis was present; therefore, we decided to perform a palliative duodenojejunal bypass. Post-operative course was uneventful, after an oral contrast CT the patient was discharged on IX POD. Unfortunately, the patient passed away 3 months after the palliative procedure.

## DISCUSSION

SBA represents a clinical challenge, due to its heterogeneous and vague behavior. The patients usually seek medical attention lamenting non-specific symptoms such as abdominal pain, cramping or bloating. A positive fecal occult blood test, melena or anemia can be already present at the time of diagnosis [1]. Frequently, the patient is referred to emergency department with small bowel obstruction or major GI bleeding [2].

CT scan accuracy is still limited. SBA is at times suspected intraoperatively and eventually confirmed with histological examination. Imaging reveals a metastatic disease in one-third of the patients [3].

Surgical approach should be considered mandatory for resectable stage I-III SBA, preceded by a thorough examination of the abdomen to rule out carcinomatosis. Nevertheless, more than 30% of treated patients develop a disease recurrence, with a disease-free survival of approximately 2 years [4]. Type of surgical treatment should be location-related: a duodenal or ileal resection with a lymphadenectomy harvesting at least 8 regional lymph nodes, or a pancreaticoduodenectomy for tumors arising from the second portion of the duodenum. Duodenal resection for tumors arising from the third or fourth portion of the duodenum is still disputed [1].

Stage IV disease treatment indications are still debated. Radical surgery comprehending hepatic metastasectomy [5] or cytoreductive surgery with intraperitoneal chemotherapy [6] were reported to be feasible and effective. CRS + HIPEC is reported to have mixed results in terms of beneficial role considering OS and DFS compared with palliative systemic chemotherapy [7–9].

## CONCLUSION

SBA can be considered one of the bugbears of gastrointestinal tumors, due to low prevalence, lack of screening program the exiguous therapeutic opportunities and the advanced stage at diagnosis.

Surgical resection with local lymphadenectomy is mandatory in stage I-III, despite a high rate of local recurrence.

To this day, CRS and HIPEC still lack evidence of efficacy. Pressurized intraperitoneal aerosol chemotherapy could be considered for palliative care, despite scarcity of clinical trials.
